# Estimation of Heart Rate Variability Parameters by Machine Learning Approaches Applied to Facial Infrared Thermal Imaging

**DOI:** 10.3389/fcvm.2022.893374

**Published:** 2022-05-17

**Authors:** Andrea Di Credico, David Perpetuini, Pascal Izzicupo, Giulia Gaggi, Daniela Cardone, Chiara Filippini, Arcangelo Merla, Barbara Ghinassi, Angela Di Baldassarre

**Affiliations:** ^1^Department of Medicine and Aging Sciences, University “G. d’Annunzio” of Chieti - Pescara, Chieti, Italy; ^2^Reprogramming and Cell Differentiation Lab, Center for Advanced Studies and Technology, University “G. d’Annunzio” of Chieti - Pescara, Chieti, Italy; ^3^Department of Neurosciences, Imaging and Clinical Sciences, University “G. d’Annunzio” of Chieti - Pescara, Chieti, Italy; ^4^Department of Engineering and Geology, University “G. d’Annunzio” of Chieti - Pescara, Chieti, Italy

**Keywords:** heart rate variability, cardiovascular risk assessment, infrared thermography, machine learning, remote sensing, support vector machine

## Abstract

Heart rate variability (HRV) is a reliable tool for the evaluation of several physiological factors modulating the heart rate (HR). Importantly, variations of HRV parameters may be indicative of cardiac diseases and altered psychophysiological conditions. Recently, several studies focused on procedures for contactless HR measurements from facial videos. However, the performances of these methods decrease when illumination is poor. Infrared thermography (IRT) could be useful to overcome this limitation. In fact, IRT can measure the infrared radiations emitted by the skin, working properly even in no visible light illumination conditions. This study investigated the capability of facial IRT to estimate HRV parameters through a face tracking algorithm and a cross-validated machine learning approach, employing photoplethysmography (PPG) as the gold standard for the HR evaluation. The results demonstrated a good capability of facial IRT in estimating HRV parameters. Particularly, strong correlations between the estimated and measured HR (*r* = 0.7), RR intervals (*r* = 0.67), TINN (*r* = 0.71), and pNN50 (%) (*r* = 0.70) were found, whereas moderate correlations for RMSSD (*r* = 0.58), SDNN (*r* = 0.44), and LF/HF (*r* = 0.48) were discovered. The proposed procedure allows for a contactless estimation of the HRV that could be beneficial for evaluating both cardiac and general health status in subjects or conditions where contact probe sensors cannot be used.

## Introduction

Heart rate variability (HRV) is the temporal variation of the period between consecutive heartbeats, which mainly depends on the regulation of the heart rate (HR). HRV analysis allows for assessing overall cardiac health and the condition of the autonomic nervous system (ANS) (1, 2). Specifically, HRV reflects neurocardiac function since it is generated by the interaction between the heart and dynamic nonlinear ANS processes; particularly, the HR is dictated by the balance between the sympathetic nervous system (SNS) and parasympathetic nervous system (PNS): dominance of the SNS activity with respect to that of the PNS produces an acceleration of the cardiac rhythm, whereas a prevalence of the PNS activation causes a deceleration of the HR. Hence, the variability in the HR is indicative of the functioning of the autonomic nervous control on the cardiac rhythm and the heart’s responsiveness (3). Moreover, a link between cardiovascular state and HRV was found (4–6), fostering both technological advancements for HRV measurement and improvement of the effectiveness of HRV data analysis to predict cardiovascular and psychophysiological states. The ANS state is linked with a variety of diseases (7–9); in fact, HRV is considered an important diagnostic tool in cardiovascular clinical practice and can be used to estimate the integrity of cardiac autonomic innervation and the vulnerability to cardiac arrhythmias resulting from autonomic imbalance (9). It has been demonstrated that HRV can be used for the risk assessment in patients recovering from myocardial infarction (10). In addition, several HRV indices have a high prognostic value to identify patients at risk for cardiovascular events (11). Furthermore, impaired HRV can be used to predict the risk of arrhythmic events after acute myocardial infarction, assess diabetic neuropathy (8), mitral valve abnormalities (12, 13), and study inappropriate sinus tachycardia or postural orthostatic tachycardia syndrome (POTS) to determine if there is evidence of parasympathetic dysfunction as a cause of the condition (14). In addition, HRV is also widely used in the exercise field to monitor the autonomic and cardiovascular conditions of the all-age population, and this is a crucial point since the cardiometabolic risk is increasing in the general population other than people with well-known risk factors (15–17).

Wearable devices were developed to provide continuous monitoring of the heart rhythm through photoplethysmography (PPG) (18–21). PPG is an optical technique able to estimate HRV by means of the pulse rate variability (PRV) (22, 23). In particular, PPG is sensitive to hemoglobin variations in vessels, which are due to volumetric changes of peripheral arteries in response to the propagation of pulse pressure waves from the heart (18). Concerning data analysis, several indices have been proposed in order to assess variations in HR in both the time and frequency domains.

For instance, the root mean square of the successive differences (RMSSD) of the neighboring ECG R-peaks (R–R intervals) is a time-domain feature sensitive to the ANS’s parasympathetic branch (24). The RMSSD reflects the variance between consecutive heartbeats and is one of the most used time-domain measures to obtain the estimation of the PNS-mediated changes observed in HRV (25). Another used time domain is the standard deviation (SD) of the interbeat intervals of normal-to-normal RR intervals (SDNN, measured in ms), where “normal” means that atypical heartbeats (e.g., ectopic beats) have been eliminated. The percentage of adjacent NN intervals that differ from each other by more than 50 ms (pNN50 %) is a time-domain parameter, which can be considered analyzing 60 s period of recording. This is highly correlated with parasympathetic activity and may be a reliable index for brief samples used in biofeedback settings. In addition, the triangular interpolation of the NN interval histogram (TINN) represents the baseline width of a histogram showing the NN intervals (2, 26).

Concerning the frequency domain, the ratio between power spectrum densities (PSDs) at low-frequency (LF, 0.03–0.15 Hz) and high-frequency (HF, 0.15–0.35 Hz) bands of the HRV is commonly employed (27). Specifically, PNS contributes to HF power, whereas LF is related to both SNS and PNS, hence, their ratio (LF/HF) is indicative of the balance between the two systems (2, 27–29).

Typically, HRV is evaluated using methods that require skin contact (e.g., electrocardiography, ECG, and PPG), but, although they are noninvasive, the contact with human skin can be detrimental to subjects with sensitive skin (e.g., neonates and patients with skin injury) and irritating or distracting when worn, for instance, in a professional environment. In these scenarios, measuring HRV through contactless technology would be beneficial. To overcome this limitation, several methods have been developed to assess HRV without skin contact. These techniques are commonly referred to as remote photoplethysmography (rPPG), and they exploit models based on red, green, and blue (RGB) imaging to assess periodic variation of the subject’s skin color, which depends on the cardiac cycle (30, 31). These methods allow for the monitoring of the health and psychophysiological conditions of individuals during several tasks in a completely ecological manner, improving human safety and wellbeing. However, scarce illumination conditions could be detrimental to these methods, degrading the performance of the HRV assessment. Infrared thermography (IRT) could be employed to overcome this limitation. IRT is a noninvasive contactless technique that passively measures the radiation from a body, estimating its superficial temperature (32). Usually, facial IRT is employed to provide information on the human autonomic activity, considering the temperature time course and spatial distribution of responsive region of interest (ROIs) (33, 34).

Recently, machine learning (ML) frameworks have been used to increase the capability of IRT in detecting pathologies and modulations of autonomic activity (35, 36).

The aim of this study is to investigate the feasibility of estimating HRV parameters from facial IRT through an ML approach. Specifically, a support vector regression (SVR) was implemented to estimate time and frequency domain metrics (i.e., RMSSD, SDDN, pNN50 %, TINN, and LF/HF) and the average HR and RR intervals from features evaluated on the temperature time course of facial ROIs.

Specifically, the model does not require inference of the physiology of the system, but the only *a priori* hypothesis was the presence of a physiological link between HRV and skin temperature modulations. This assumption was tested *a posteriori* with the assessment of the generalization performance of the model, through a cross-validation approach. This model is useful for remote sensing of health conditions, which is fundamental to monitor patients’ improvements during rehabilitation and therapies (37). Moreover, during the COVID-19 pandemic, several solutions have been proposed in order to remotely assess the vital signs and the clinical conditions of patients to avoid the contagious (38). Furthermore, contactless monitoring of the emotional and autonomic conditions can be used to monitor cardiovascular risk and patient’s prognosis in clinical practice when methods that require skin contact are not possible to implement and for individuals performing tasks, such as working and driving, improving the human safety and wellbeing.

## Materials and Methods

### Experimental Procedure and Data Acquisition

The experimental session involved 32 healthy volunteers (20 women and 12 men, age = 51.46 ± 7.68 years). The paradigm induced modifications in the HRV of the participants through modulations of the breathing rate and intensity (39). In detail, participants had to perform a breathing task comfortably sitting on a chair in front of a computer staring at a visual stimulus presented on the screen of the computer modulating the rate and the intensity of their breathing. The experimental session lasted 1 min. To collect information on the pulse rate variability of the participants, a PPG sensor (emWave Pro Plus, HeartMath, Inc., Boulder Creek, CA, United States) was placed on the fingertip of the subjects’ left hand during the task. The sampling frequency was 370 Hz.

Concurrently, the facial temperature was recorded through a digital thermal infrared camera FLIR SC660 (FLIR, Wilsonville, OR, United States) (640 × 480 bolometer FPA, sensitivity/noise equivalent temperature difference: <30 mK at 30°C, the field of view: 24° × 18°). The IRT device was placed 60 cm distant from the participant and pointed toward his/her face. The sampling frequency was 10 Hz. The camera was blackbody-calibrated in order to eliminate eventual drift/shift of the sensor’s response and optical artifacts.

The study was approved by the Research Ethics Board of the University of Chieti-Pescara (approval number: 1479, date of approval: 03/05/2017), and it followed the principles of the Declaration of Helsinki. Each participant signed the informed consent, and they could withdraw from the experiment at any time. The measurements were performed following the standard guidelines for thermal imaging acquisitions (40). Specifically, the experiment was performed in a thermoneutral environment to prevent the risk of possible thermoregulatory-induced alterations. Moreover, the participants could acclimate to the environment for a period of 15 min before the session to reach the thermal equilibrium (40). Furthermore, all the sessions were scheduled at the same time of day with the aim to avoid the effects of possible circadian rhythm variations (41).

### Data Preprocessing

Concerning PPG, the signals were band-pass filtered setting the cutoff frequencies at 0.2 and 10 Hz. A procedure for automatic peaks identification is employed for the PPG filtered and normalized (*z*-score) signals. The performance of the algorithm was checked by visual inspection, but no correction had to be applied. The PPG peaks were used to evaluate the HRV metrics over the 1 min recording. Particularly, the following indices were computed: mean HR (beats/min), mean RR (ms), LF/HF, SDDN (ms), RMSSD (ms), pNN50 (%), and TINN (ms). Table 1 reports the formulas employed to evaluate the HRV metrics considered. In Table 2, the list of thermal features extracted from each ROI selected to feed the different SVR models used to estimate the HRV parameters is reported.

**TABLE 1:** Computation of HRV indices on the PPG signals, empoyed as output of the ML models developed.

Variables	Description
HR (bpm)	Mean heart rate
RR (ms)	Mean of RR intervals
TINN (ms)	Baseline width of the RR interval histogram
pNN50 (%)	NN50 divided by the total number of RR intervals
RMSSD (ms)	Square root of the mean squared differences between successive RR intervals
SDNN (ms)	Standard deviation of NN intervals
LF/HF	Ratio between LF and HF band powers

**TABLE 2:** Thermal features extracted from each ROI selected to feed the different SVR models used to estimate the HRV indices considered.

HRV Metrics	Predictors
HR (bpm)	Mean value (NT and N), std (G, NT and N), kurtosis (G and N), skeweness (N), SampEn (G and N) and PSD-Breath (N)
RR (ms)	Mean value (NT), std (G and N), kurtosis (NT), SampEn (G and N), PSD-Breath (G) and PSD-Cardiac (NT)
TINN (ms)	Std (NT and N), kurtosis (NT), skewness (N), SampEn (G) and PSD-Myo (NT)
pNN50 (%)	Mean Value (G and N), std (G, NT and N), kurtosis (G) and PSD-Cardiac (N)
RMSSD (ms)	Mean value (G), std (G and N), kurtosis (G and NT), skewness (N), SampEn (G and N), PSD-Breath (G), PSD-Cardiac (NT and N) and PSD-Myo (NT and N)
SDNN (ms)	Std (G), kurtosis (G and NT), SampEn (G and N), percentile (G), PSD-Breath (G, NT and N)
LF/HF	Kurtosis (N), SampEn (G and N), PSD-Breath (NT and N) and PSD-Cardiac (G and NT)

*The ROI on the glabella is referred as G, the one on the nose tip is called NT, and the one on the nostrils is called N. The input features reported are obtained from the wrapper method in order to reduce the number of regressors and to optimize the models’ performance.*

**FIGURE 1:**
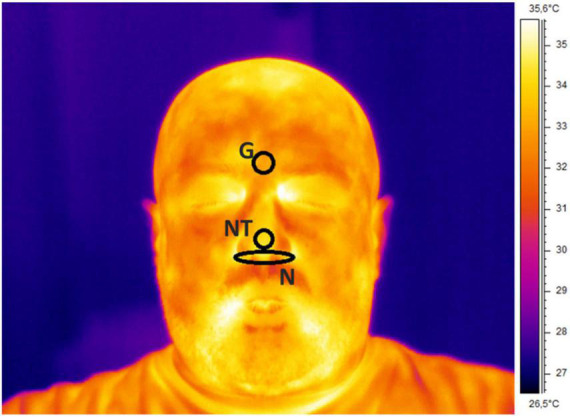
Thermogram of a representative participant with ROIs placement over the glabella (G), nose tip (NT), and nostrils (N).

Regarding the IRT recordings, the quality of the signals was checked by visual inspection and no video was rejected. Three ROIs were selected on the glabella (G), nose tip (NT), and nostrils (N). Since the participants stayed quite still during the experiment, the algorithm did not fail in tracking any frames. The following features were computed from the temperature time course of each ROI to feed the machineries: mean value, SD, kurtosis, skewness, variation of the signal computed as the difference between the average of the first and last 5 s of the acquisition (delta), sample entropy (SampEn), 75° percentile, the power spectra density (PSD) of the thermal signal for the respiratory (PSD-breath), cardiac (PSD-cardiac), and myogenic (PSD-myo) frequency bands. In detail, mean value, SD, kurtosis, and skewness are different moments of the distributions, providing information on the central tendency, dispersion, and shape of the temporal evolution of the temperature. Concerning delta and 75° percentile, they are parameters indicative of the variability of the signal. The sample entropy is a measure of the predictability of the signals, which is defined as the negative natural logarithm of the conditional probability that signal subseries of length m (pattern length) that match pointwise within a tolerance r (similarity factor) also match at the m+1 point, and it evaluates nonlinear predictability of the signal (42). The PSD describes the distribution of power into frequency components composing that signal. In this study, the mean value of the PSD evaluated over the following frequency bands was computed: myogenic band (0.04–0.15 Hz), respiratory band (0.15–0.5 Hz), and cardiac band (0.5–1 Hz) (43). The frequency bands were considered. Of note, all the features were normalized (*z*-score) before being employed in the ML framework. A representative thermogram of one participant is reported in Figure 1.

### Machine Learning Procedure

An SVR approach was used as previously described (44). In this case, the SVR-based models with linear kernel were developed to predict the different HRV metrics [i.e., mean HR (beats/min), mean RR (ms), LF/HF, SDDN (ms), RMSSD (ms), pNN50 (%), and TINN (ms)] evaluated from the PPG signals. The features extracted from the temperature time course of each ROI were used as input for the SVR models. Of note, since the number of predictors (i.e., number of ROIs × number of features = 30) was similar to the number of participants (i.e., 32), a subset of the features was employed as an input of the ML framework, after a selection based on wrapper method (45). A fivefold cross-validation was implemented to reduce the risk of the overfitting effect and assess the generalization performance of the models. To test the stability of the results and to avoid a possible effect related to the definition of the folds, 1,000 random combinations of folds were tested in a bootstrap procedure. The models associated with the average performance assessed by the bootstrap were used for further analysis. The performance of the models was evaluated by correlation (Spearman) analysis, Bland–Altman plot, and paired *t*-test. Data preprocessing and analysis were performed using MATLAB (MathWorks, Inc., Natick, MA, United States).

## Results

**FIGURE 2:**
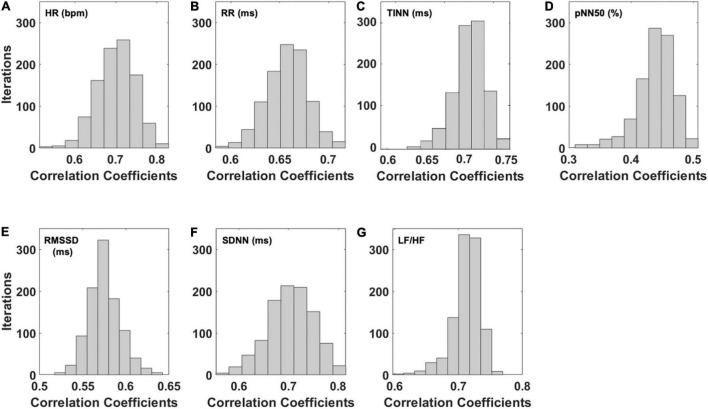
Distribution of the correlation coefficients for the predicted HRV variables after 1,000 iterations of the bootstrap procedure. In detail, the distributions of the correlation coefficients obtained for the **(A)** heart rate (HR), **(B)** interval between consecutive R peaks (RR), **(C)** triangular interpolation of the NN interval histogram (TINN), **(D)** pNN50 (%), **(E)** root mean square of the successive differences (RMSSD), **(F)** standard deviation of NN intervals (SDNN), and **(G)** ratio of LF-to-HF power (LF/HF) are shown.

Concerning the correlation analysis results, Figure 2 reports the results of the bootstrap procedure. Specifically, it reports the histogram of the distribution of the correlation coefficients obtained after 1,000 iterations during which the folds were randomly chosen. Results showed a strong correlation regarding HR (Figure 2A; *r* = 0.70), RR (Figure 2B; *r* = 0.66), TINN (Figure 2C; *r* = 0.71), and pNN50% (Figure 2D; *r* = 0.70), while a moderate correlation was found for SDNN (Figure 2E; *r* = 0.44) and LF/HF (Figure 2F; *r* = 0.48).

To summarize the data and show the stability of the model, the average and associated SD of the correlation coefficients delivered by the bootstrap analysis for all the HRV metrics are reported in Table 3.

**TABLE 3:** Mean values of the correlation coefficients (r) and associated standard deviation were obtained through the bootstrap procedure for each HRV metric estimated.

Variables	Average r ±SD	r^2^	Regression equation
HR (bpm)	0.70 ± 0.04	0.48	y = 0.93x + 3.12
RR (ms)	0.66 ± 0.02	0.43	y = 0.93x + 38.7
TINN (ms)	0.71 ± 0.02	0.51	y = 0.30x +179
pNN50 (%)	0.70 ± 0.05	0.50	y = 0.68x + 5
RMSSD (ms)	0.58 ± 0.02	0.35	y = 0.59x + 19.5
SDNN (ms)	0.44 ± 0.03	0.19	y = 0.46x + 25.9
LF/HF	0.48 ± 0.03	0.23	y = 0.46x + 28.4

*Additionally, r^2^ and regression equations are reported.*

**FIGURE 3:**
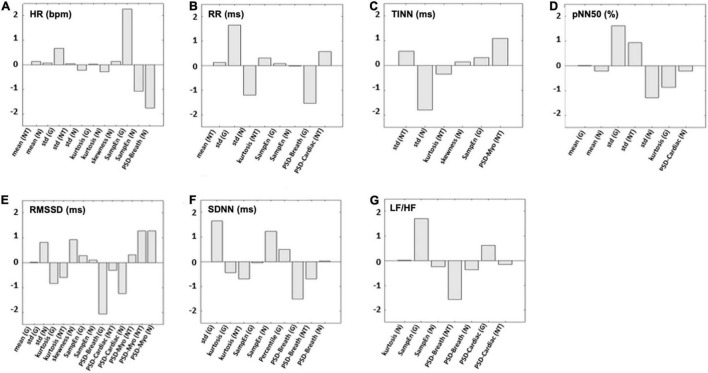
The weights for each predictor used in the support vector regressor (SVR) models to estimate **(A)** HR, **(B)** RR, **(C)** TINN, **(D)** pNN50 (%), **(E)** RMSSD, **(F)** SDNN, and **(G)** LF/HF are shown. N, nostrils; NT, nose tip; G, glabella.

The models associated with the average correlation coefficient obtained with the bootstrap procedure were used for further analysis. Figure 3 reports the weights of the cross-validated models developed for each HRV indices estimated. SampEn measured at the glabella (G) showed the highest weight for the prediction of HR and LH/HF (Figures 3A,G). The SD measured at the G had the highest predictive value for RR, pNN50 (%), and SDNN (Figures 3B,D,F). Power spectrum density of the thermal signal for the myogenic frequency band (PSD-myo) measured at the NT had the highest predictive value for both TINN and RMSSD (Figures 3C,E). Regarding this latter, also PSD-myo measured at the N showed a weight as high as that measured at the NT (Figure 3E).

**FIGURE 4:**
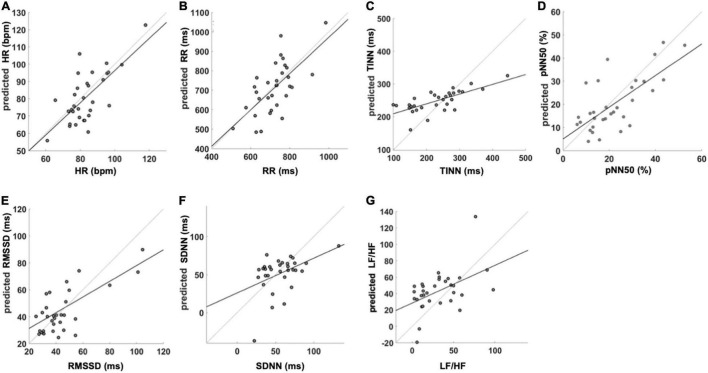
SVR performance for the analyzed HRV variables. The figure reports the regression equation and *r*^2^ for the prediction of **(A)** HR, **(B)** RR, **(C)** TINN, **(D)** pNN50 (%), **(E)** RMSSD, **(F)** SDNN, and **(G)** LF/HF. HR, heart rate; RR, the interval between consecutive R peaks; pNN50 (%), percentage of successive RR intervals that differ by more than 50 ms; RMSSD, root mean square of successive RR interval differences; SDNN, standard deviation of NN intervals; LF/HF, ratio of LF-to-HF power. The reported values are associated with the iteration closer to the mean correlation coefficient.

In addition, Figure 4 reports the correlation plot obtained between the measured HRV indices and the ones predicted form IRT signal features. Importantly, high coefficients of determination (*r*^2^) were found for HR (Figure 4A; *r*^2^ = 0.48), RR (Figure 4B; *r*^2^ = 0.43), TINN (Figure 4C; *r*^2^ = 0.51), pNN50 (%) (Figure 4D; *r*^2^ = 0.50), and RMSSD (Figure 4E; *r*^2^ = 0.35). Moderate to weak *r*^2^ values were found for LF/HF (Figure 4G; *r*^2^ = 0.23) and SDNN (Figure 4F; *r*^2^ = 0.19), respectively.

Table 4 reports the results of the paired *t*-test between the measured HRV metrics and those predicted from IRT. No significant results were showed for HR (*t* = 1.02, *p* = 0.317), RR (*t* = 0.55, *p* = 0.583), TINN (*t* = –1.75, *p* = 0.090), pNN50 (%) (*t* = –1.26, *p* = 0.220), RMSSD (*t* = 1.18, *p* = 0.248), and SDNN (*t* = 1.08, *p* = 0.289), indicating no differences between the measured and predicted values. Conversely, a significant difference was highlighted for LH/HF (−2.43, *p* = 0.021).

**TABLE 4:** Results of the *t*-test were computed to compare the HRV metrics evaluated from the PPG signal and the correspondent metrics estimated by the cross-validated SVR.

Variables	t-stat	df	*p*-value
HR (bpm)	1.02	31	0.317
RR (ms)	0.55	31	0.583
TINN (ms)	–1.75	31	0.090
pNN50 (%)	–1.26	31	0.220
RMSSD (ms)	1.18	31	0.248
SDNN (ms)	1.08	31	0.289
LF/HF	–2.43	31	0.021

**FIGURE 5:**
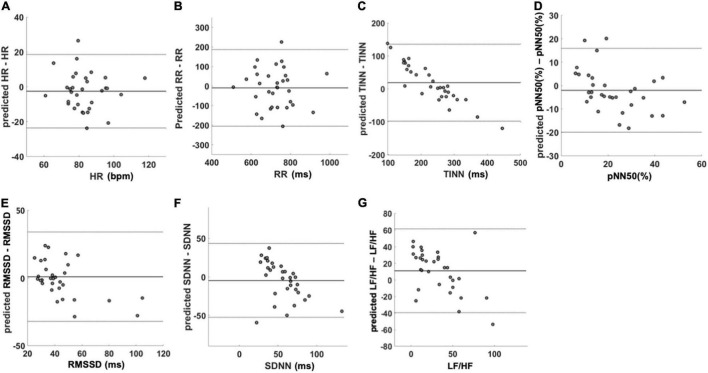
Bland–Altman plots of the predicted HRV variables. The figure shows the agreement between the variables measured with PPG and the predicted ones using IRT data. In each figure, mean difference and limit of agreement are reported. **(A)** HR, **(B)** RR, **(C)** TINN, **(D)** pNN50 (%), **(E)** RMSSD, **(F)** SDNN, and **(G)** LF/HF. HR, heart rate; RR, interval between consecutive R peaks; pNN50 (%), percentage of successive RR intervals that differ by more than 50 ms; RMSSD, root mean square of successive RR interval differences; SDNN, standard deviation of NN intervals; LF/HF, ratio of LF-to-HF power.

Concerning the agreement between measured and predicted HRV variables, the Bland–Altman plot for HR (Figure 5A) showed a mean difference of −2.6 beats/min (*p* = 0.19) with a lower limit of agreement (LoA) of −24.0 beats/min and an upper LoA of 19.0 beats/min. Regarding the RR intervals (Figure 5B), the mean difference was −9.8 ms (*p* = 0.58) with a lower LoA of −210.0 ms and an upper LoA of 190.0 ms. The mean difference for the TINN (Figure 5C) accounted for 18.0 ms (*p* = 0.09) with a lower LoA of −98.0 ms and an upper LoA of 140.0 ms. Concerning the pNN50 (%) (Figure 5D), the mean difference was −2.0 with a lower and upper LoA of −20.0 and 16.0, respectively. The RMSSD (Figure 5E) showed a mean difference of 1.0 ms (p = 0.73) with a lower LoA −32.0 ms and an upper LoA of 34.0 ms. The mean difference for SDNN (Figure 5F) was −4.7 ms (*p* = 0.29) while lower and upper LoA were −53.0 and 43.0 ms, respectively. Finally, the mean difference for the LF/HF was 11 (*p* = 0.02) with a lower LoA of −39.0 and an upper LoA of 61.0 (Figure 5G).

## Discussion

This study investigated the possibility of estimating HRV parameters from facial temperature skin oscillations by developing models based on SVR with a linear kernel. To test the generalization performance of the models, a five-fold cross-validation approach was implemented. Furthermore, to prevent a possible overfitting effect due to the choice of the folds used to train and test the model, a bootstrap procedure with 1,000 iterations was performed for each model. The performance of the models was tested through correlation analysis to investigate the relationship between the real and predicted metrics. To assess bias between the measured and estimated indices, the *t*-test was used, and the Bland–Altmann plot was implemented to evaluate the agreement between the PPG- and IRT-derived parameters. In accordance with Cohen’s interpretation of the correlation coefficient (28), a strong correlation between the estimated and measured metric was found for the average HR, the average RR intervals, pNN50 (%), and TINN, whereas a moderate correlation was found for RMSSD, SDNN, and LF/HF. To show the average behavior of the model and its stability, the mean value and SD of the distributions of the correlation coefficients obtained for the iterations were reported. Of note, although these metrics are more suitable to describe the normal distribution, they are able to provide information about the reliability and robustness of the performance of the model. Accordingly, data highlighted that a possible overfitting effect due to the creation of the folds is not present. Regarding the presence of a bias in the estimation of the HRV metrics, the paired *t*-tests assessed significant differences for LF/HF between the estimated and measured metrics. Notably, concerning TINN, the *t*-test delivered a *p*-value close to the chosen significance (*p* < 0.05), whereas the other metrics did not exhibit a bias when estimated from IRT signals features. Regarding the Bland–Altman plot results, a clear systematic error of the model is assessed for LF/HF, SDNN, and TINN. Considering the linear regression equation associated with each model to estimate the different HRV metrics, it is worth to highlight that a good model should deliver a linear equation with the slope around 1 and the constant term around 0 (46). The models implemented delivered a slope above 0.5 for HR, RR, pNN50 (%), and RMSSD. Concerning the constant terms, they are above 0 for all the models, allowing us to obtain an estimation without bias (assessed by the *t*-test) of almost all the HRV metrics, although the slope is not close to 1. However, these features of the regression equation produce the systematic error assessed for some models.

Considering all these aspects, the findings demonstrated the feasibility to develop an acceptable model from IRT data for HR, RR, pNN50 (%), and RMSSD.

The employment of a model with a linear kernel allows to establish clear relationships between the predictors used as input and the output of the machinery through the analysis of the weights associated with each regressor (47). Moreover, the linear kernel is less prone to overfitting effects with a limited dataset (48). On the contrary, introducing a nonlinearity in the prediction is not always beneficial to the performance. In fact, if the relationship between the input regressors and the output is well expressed by linear regression, using nonlinearities could be detrimental to the performance of the model. Concerning the HR model, the predictors exhibiting the highest weights are the SampEn evaluated on the G and N and the PSD-breath evaluated on the N. Of note, the negative sign of SampEn (N) and PSD-breath (N) implies an inverse relationship between the HR and these regressors, whereas the SampEn (G) is positively related to this HRV metric. Regarding the RR model, the highest weights of the regression are associated with the SD of the signal evaluated on the G and N and the PSD-breath computed on the G. Of note, the weight associated with SD (G) is positive, whereas the ones associated with SD (N) and PSD-breath (G) are negative. In the model developed for pNN50 (%), the largest positive weights are associated with SD computed over the G and NT, whereas the largest negative weights are related to SD of the thermal signals measured on the N and to the kurtosis of the signal collected from the G. Finally, the RMSSD model shows a predominance of the weights associated with the PSD. Particularly, positive weights are associated with PSD evaluated over the myogenic band for the NT and N, whereas PSD-breath (G) and PSD-cardiac (N) exhibit negative weights. From these results, it is evident that the estimation of the HRV indices is mainly due to metrics of variability (SD) and predictability (SampEn) of the signal with respect to metrics of central tendency (mean value). This finding suggests that the HRV can produce variations of the thermal signal. In fact, modulations of the HR can produce oscillations of the blood volume of the vessels, which is related to the skin temperature variations (49). This assumption is also confirmed by the important role of the PSD evaluated over the cardiac frequency band for estimating the RMSSD. Moreover, the contribution of the PSD over the myogenic band in the estimation of the RMSSD could be explained by the influence of the vasomotor regulation of the vessels on the skin temperature (50). Finally, the importance of the respiratory band could be related to the influence of the breathing rate on both HR and HRV (51) but also to the relevance of this physiological process in the ROIs considered. In fact, the temperature time course of the NT and N is strongly influenced by the modulations of the breath, since the inspired air has a different temperature with respect to the exhaled air (52). Of note, the acquisitions have been performed during the execution of a breathing task, further confirming the assessed importance of these features in the estimation of HRV indices. The findings of this study are based on a strong relationship between HRV and facial thermography. In fact, it is well known that both HRV and facial temperature time courses are indicative of the psychophysiological condition of the individual, and they are both influenced by the ANS activity (53, 54). Particularly, IRT can provide information regarding the superficial circulation, which is modulated by the ANS, responsible for thermoregulation processes (55). Particularly, the venous return and the cardiac output are also modulated by the heat transfer from skin to blood, and subsequently to the body core (56). However, it is worth to highlight that the superficial microcirculation is influenced also by local factors, hence, it could be licit to suppose that the not optimal correspondence between the estimated and measured HRV metrics is related to different physiological mechanisms underlying the cardiac rhythm and the skin temperature modulations (57). In addition, it is useful to highlight that PPG signals could vary based on the measurement site and the skin temperature. Mejía-Mejía et al. showed that the correlation between HRV measured through PPG and that measured with ECG can slightly change based on the recording site, and regarding the fingertip, they found a high correlation (58). Accordingly, Selvaraj and colleagues found a correlation of 0.97 between ECG-derived RR intervals and PPG-derived peak-to-peak intervals (59). Thus, the finger was chosen as the recording site since it is considered the gold standard for clinical PPG acquisitions (60). In addition, the effect of temperature was controlled allowing the participant to acclimate to the environment for a period of 15 min, to reach the thermal equilibrium (61). In this way, pulse rate variability was measured in normothermia conditions for every participant, thus preventing a possible impairment of the method’s performance due to the temperature variability.

The proposed procedure could represent a valuable tool for continuous HRV monitoring also in poor environmental light conditions. The employment of IRT for HRV monitoring also allows to concurrently evaluate other physiological signals, such as breathing rate and sweat glands activity. These signals are indicative of the emotional status of the individual and can provide information on the wellbeing of the subject. This procedure could be highly suitable for clinical applications, such as bedside vital signs monitoring, particularly for those patients where contactless monitoring is preferred. The possibility to use IRT to predict HRV parameters is of main importance to investigate the health status of subjects with skin diseases, skin injury, and neonates. In fact, HRV values are associated with the risk, onset, and prognosis of a plethora of diseases. The short-term HRV recordings show that low values of RMSSD and SDNN were associated with an increased risk of coronary heart disease and death from different causes (62).

Moreover, it could be employed to remotely assess the vital signs and the clinical conditions of patients, especially contagious patients, as during the COVID-19 pandemic (38). Furthermore, IRT could, in fact, be used to monitor the human psychophysiological state, stress, and emotional state in the workplace and during the execution of cognitively demanding tasks.

However, it is worth to highlight that the proposed procedure does not provide a highly accurate estimation of all the HRV metrics, and hence it could be worth to perform further studies to improve the accuracy of the method. In fact, the first limitation of the study is represented by the reduced number of participants. Enlarging the sample size could allow to obtain more accurate results and test more complex machineries, such as deep learning. However, the proposed model was developed employing a cross-validation procedure, hence guaranteeing the generalization performance of the model.

Moreover, further studies are necessary to test the capability of estimating other HRV metrics from the facial temperature time course, also considering other ROIs or considering the whole face temperature oscillations. In addition, further studies should be performed in order to test the capability of IRT to estimate HRV metrics through ML and deep learning approaches also during resting-state condition, without the administration of a breathing task. Furthermore, the developed model can estimate HRV metrics integrating information over a temporal window of 1 min. In fact, further studies are needed to decrease the amplitude of the temporal window for the HRV prediction. Finally, it could be worth to test the feasibility to estimate the cardiac pulse signal itself, providing a valuable tool able to deliver performances similar to rPPG also in poor light conditions.

Although preliminary, these results could pave the way to the employment of IRT for contactless HRV monitoring for cardiovascular medicine and neuroergonomic applications, to be exploited, for instance, in an Internet of Things context. Specifically, this model could be suitable for clinical applications when contact technique is not suitable (e.g., patients with sensitive skin), for human–machine interaction (HMI), automotive systems, and for monitoring the wellbeing of workers in the workplace.

## Conclusion

This study proposed an innovative model based on linear SVR to estimate HRV metrics from facial temperature time course assessed by IRT. Specifically, ROIs from G, NT, and N were considered to compute thermal features used for the HRV indices prediction. The performances of the model were tested through correlation analysis, Bland–Altman plot, and paired *t*-test. Models delivering a good estimation of HR, RR, pNN50 (%), and RMSSD on a temporal window of 1 min were implemented. These results could pave the way to the employment of IRT for a contactless HRV assessment suitable for cardiovascular medicine and neuroergonomic applications also with poor light conditions or in a situation in which contact methods are not implementable. Thus, our proposed method represents a new useful tool in the field of cardiovascular medicine in different situations in which methods that require skin contact are not possible to implement.

## Data Availability Statement

The original contributions presented in the study are included in the article, further inquiries can be directed to the corresponding author.

## Ethics Statement

The studies involving human participants were reviewed and approved by Research Ethics Board of the University of Chieti - Pescara - approval number: 1479, date of approval: 03/05/2017. The patients/participants provided their written informed consent to participate in this study. Written informed consent was obtained from the individual(s) for the publication of any potentially identifiable images or data included in this article.

## Author Contributions

ADC and DP: concept, design, analysis, and drafting of the study. GG, PI, DC, and CF: drafting. AM: revision. ADB and BG: revision, funding, and project administration. All authors contributed to the article and approved the submitted version.

## Conflict of Interest

The authors declare that the research was conducted in the absence of any commercial or financial relationships that could be construed as a potential conflict of interest.

## Publisher’s Note

All claims expressed in this article are solely those of the authors and do not necessarily represent those of their affiliated organizations, or those of the publisher, the editors and the reviewers. Any product that may be evaluated in this article, or claim that may be made by its manufacturer, is not guaranteed or endorsed by the publisher.
